# Interactive Data-Gathering Posters as a Research Tool: A Case Study Assessing Public Opinion on Incorporation of Natural Behavior into Management Systems

**DOI:** 10.3390/ani10060971

**Published:** 2020-06-03

**Authors:** Mike King, James Webster, Catherine Cameron, Gosia Zobel

**Affiliations:** 1 Otago Bioethics Centre, University of Otago Medical School, Dunedin PO Box 913, New Zealand; mike.king@otago.ac.nz; 2 AgResearch Ltd., Ruakura Research Centre, Hamilton 3240, New Zealand; jim.webster@agresearch.co.nz (J.W.); Catherine.Cameron2@agresearch.co.nz (C.C.)

**Keywords:** conference techniques, presentation, poster, goat, welfare

## Abstract

**Simple Summary:**

An innovative form of poster presentation was developed and piloted, with the aim of generating data rather than just presenting it. This tool was then used with three different stakeholder groups (an international conference, a regional veterinary conference, and a regional school leadership day) to gather participant opinion on an important component of animal welfare (naturalness). The poster promoted interaction between attendees and the poster presenter via ranking stickers and allowed participants to provide direct input about their top three areas of importance regarding the topic. We demonstrated a proof-of-concept, therefore, did we not make comparisons across cohorts; however, we showed that when applied in different settings, the poster gathered some consistent opinions on which behaviors are the best exemplars of naturalness in goats. While we identified that response bias and sampling bias could both be issues with the type of interaction promoted by this tool, we suggest that corrections used in other traditional data gathering methods (e.g., focus groups) could be applied to alleviate these biases. The flexibility of this interactive tool, and its capability to shift the audience from being the viewer of, to the interactive participant in, research, presents a novel alternative to traditional poster presentations.

**Abstract:**

We developed a simple, interactive poster design. Via brief infographics and simple numbered stickers, participants were able to provide input about their top three areas of importance regarding a specific topic (i.e., promoting natural behavior in goats). The tool was utilized in three scenarios—an international conference, a regional veterinary conference, and a regional school leadership day. After a short discussion with the presenter, participants ranked their top three areas of importance. Response rates ranged from 22% to 100%. The data collection performed was intended to demonstrate a proof-of-concept of the poster design; therefore, comparisons across tested cohorts were not made. However, we showed that when applied in different settings, the poster gathered some consistent opinions on which behaviors are the best exemplars of naturalness in goats. Response bias, from opting for socially desirable responses, as well as sampling bias from using the tool at specific conferences or with specific demographics, could be an issue. Nonetheless, these are not unique concerns, and we suggest that corrections used in focus groups could alleviate these biases. The flexibility of this interactive tool, and its capability to shift the audience from viewing to participating in research presents a novel alternative to traditional poster presentations.

## 1. Introduction

The academic poster constitutes an established genre of research communication, and, as such, it has norms. Three essential elements of the poster are the presentation of scientific information on a board, an audience, and at certain times, a presenter [1]. The audience reads the poster and views its imagery, and through these actions, information is conveyed. If the presenter is present, the poster is intended to promote dialogue, forming another medium for the transfer of information.

Our approach to poster presentation alters the function of the poster radically. The normal poster provides primarily unidirectional transfer of research results from the researcher to the audience (aside from the bidirectional exchange of information that can occur in the discussion; however, this lacks standardized recording opportunity). Our poster largely inverts this format. Background information is presented as a stimulus for eliciting responses from the audience; these responses are ‘recorded’, and this process is not reliant on the presence of the presenter. The data can be used to test a research hypothesis, or to poll opinion, thus informing further inquiry (e.g., public opinion data seeding research [2,3,4]). The role of the audience shifts from the viewer of, to an interactive participant in, the research; this is important as passivity reduces poster effectiveness of information transfer (e.g., minimized engagement reduces information recall [5]).

We piloted this poster method as a means of gathering stakeholder opinion and, thus informing, our future research on dairy goat welfare. The expression of natural behavior is one of the three key concerns of animal welfare [6,7]. Naturalness is entrenched in the ‘Five Freedoms’ proposed in the Brambell report (where it is described as ‘normal’ behavior) [8]; it is represented in the fourth domain in the “Five Domains” framework [9] and is one of three fundamental components of Fraser’s definition of animal welfare [6,7]. Despite its importance, naturalness has not received a great deal of interest in welfare research involving commercial goats [10]. As such, we focused the poster on polling people regarding what opportunities they felt were most important for allowing goats to express natural behaviors. Indeed, the public acknowledges the importance of expression of natural behavior [11]; however, the details of what these behaviors entail are not usually at the forefront of people’s minds [12]. 

The public has a legitimate political stake in policy formation since it can affect their interests and democratic policy development depends on public views informing it through consultation [13]. Moreover, public consultation can make policy more responsive to the practical contexts it is applied within, revealing where policy may be misguided or could lead to unintended consequences [14]. Public views are, for these reasons, influential for the development of animal welfare policy [15] and should be taken into consideration by animal-use industries. Expert opinion is also important for informing animal welfare policy and animal management practices, and there is evidence that the public is willing to trust scientists to make these decisions [2]. By developing and piloting this poster with both sets of groups, we aimed to demonstrate its usefulness for gathering data in settings where traditional posters would only act as mediums of information distribution.

This research had two aims. First, we developed and piloted an innovative, yet simple, form of poster presentation, which aimed to increase dialog, and generate data rather than merely present it. Second, we used this poster technique in an animal welfare exemplar to generate indicative data representing stakeholder views; specifically, participants expressed their opinion on conditions and behaviors that would contribute positively to naturalness for farmed goats. 

## 2. Materials and Methods 

Peer review and ethical approval for this research was obtained through the Human Ethics process at AgResearch (#5/2016). 

### 2.1. Poster Design and Content

**Figure 1:**
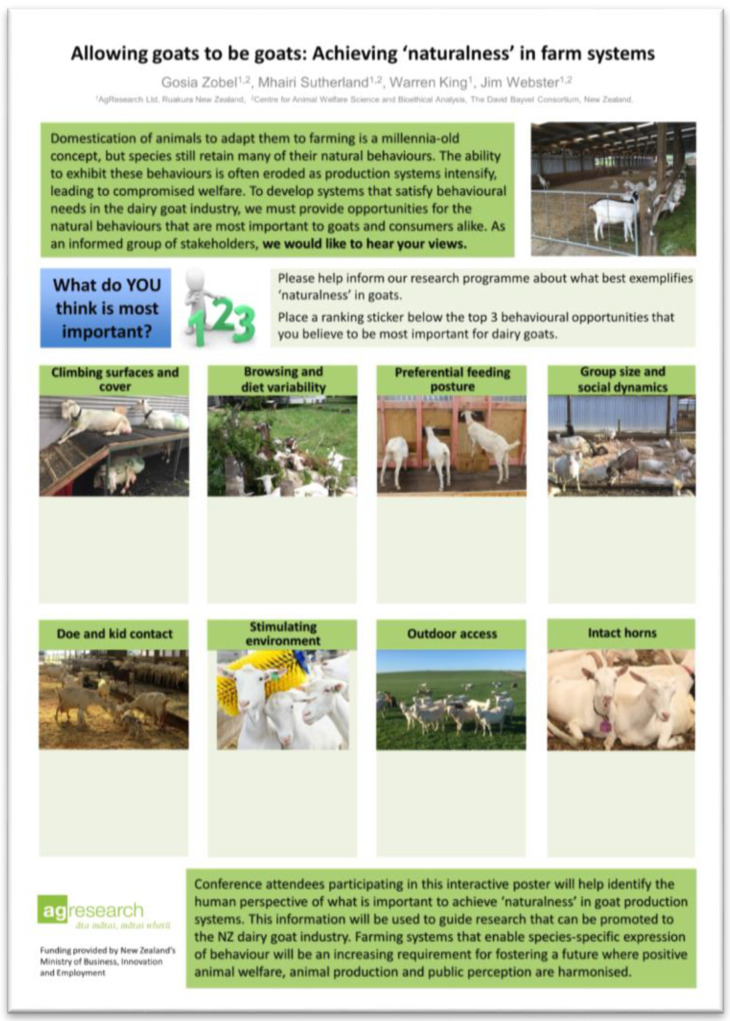
An interactive poster used to collect data from participants in various scenarios engaging stakeholders. Participants provided their opinion about which top three areas were important for promoting natural behavior in goats by placing numbered stickers in the light green boxes.

The poster was printed onto 84 cm × 119 cm durable satin fabric (Flagmakers, Wellington, New Zealand). Key components of the poster (Figure 1) included introductory information about the study aim and instructions that stated: “Please help inform our research program about what best exemplifies ‘naturalness’ in goats. Place a ranking sticker below the top three behavioral opportunities that you believe to be most important for dairy goats.” An illustrative presentation of the respondents’ task was included, as well. Initially, the potential behavioral opportunities were identified via informal discussions with our research team and with farmers. Themes identified within these discussions were considered and expanded upon to develop a broad overview of natural behavior in goats (see review [10]). From this review, we selected eight opportunities for use on the poster. Each had a photograph and they were labeled—climbing surfaces and cover, browsing and diet variability, preferential feeding posture, group size and social dynamics, doe and kid contact, stimulating environment, outdoor access, and intact horns. Respondents were asked to rank the top three opportunities according to their opinion. 

**Figure 2:**
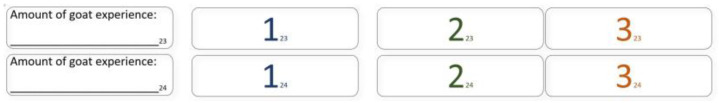
Rankings were printed onto small address labels and included a numerical identifier that allowed the research team to allocate simple demographic information to each ranking while keeping the data anonymous.

The ranking stickers were pre-printed onto labels (Removable Multi-purpose Labels L7656, Avery Ltd., Castle Hill, Australia); participants selected them from a sheet, which contained a demographic question (Figure 2) specific to the stakeholder group at each of the three events. At a research conference (World Organisation for Animal Health World Conference on Animal Welfare - OIE; 6–8 December 2016), participants were asked to report their world region and occupation. At an AgriLeadership student site visit to AgResearch Ltd. (AGRI; 17 January 2017), students were asked to indicate the type of animals with which they had the experience. At a veterinary conference (Small Ruminant Veterinarians of Ontario Annual Conference – SRVO; 7 March 2017), participants were asked to state their experience (in number of years) with goats. While encouraged to do so, participants did not have to complete all demographic information in order to participate. 

### 2.2. Poster Presentation and Participants

Prior to contributing to data collection, all participants were made aware that participation was voluntary and that no identifying information would be collected, thus anonymizing the data. At OIE, the poster was positioned in the poster viewing area, alongside traditional posters reporting scientific research results. Two of the study authors (GZ and JW) presented the poster during conference breaks and encouraged participation; however, the ranking stickers were available for participants throughout the entire duration of the conference. Stickers were removed at the end of each day. At AGRI and SRVO, the poster was hung near the front of the room as an introduction was given by GZ. Participants were then given time to allow anyone interested in completing it to do so. Following this, GZ reviewed the broad results and engaged in dialogue with the audience regarding the behavioral opportunities that received the most #1 ranking stickers and those that received the fewest stickers. At all three events, if participants commented on the poster format, these were recorded; however, they were not prompted specifically to give feedback. 

### 2.3. Data Handling

Stickers were removed from the poster following the presentation and recorded. The ranking data were first collated for each behavioral opportunity (e.g., number of 1st, 2nd, and 3rd ranking stickers placed per section). A weighted score was then calculated (Excel, Microsoft Corp., Redmond, WA, USA) for each:

(1) $$=\frac{\left(\mathrm{no}.\;1\mathrm{st}*3\right)+\left(\mathrm{no}.\;2\mathrm{nd}*2\right)+\left(\mathrm{no}.\;3\mathrm{rd}*1\right)}{6}$$

The three highest weighted scores signified the three behavioral opportunities for the participants. Weighted scores were calculated for each event’s data together, and then also according to the demographics collected at each event. When complete demographic information was not available (i.e., 17 out of 84 respondents at OIE indicated their occupation, but not their region), the ranking data were excluded when calculating weighted scores relevant to demographics but were included in the overall calculation of weighted scores. Answers were categorized according to the demographic question asked. For the OIE conference, demographics reported were region (Africa, Asia/Australia/New Zealand, Central America, EU/UK, North America, or South America), and occupation (Academic/Student, Government/NGO, Researcher, Veterinarian, or ‘Other’; the latter included farm management, consultants, trainers, and ‘trade specialist’). The AGRI students were categorized into either farm background or pet/no animals. For SRVO, demographics reported were categorized based on the number of years of experience with goats (0 to 4, 5 to 10, 11 to 20, or 21+); text-based answers were provided by four of the SRVO respondents, and these were categorized to be as representative as possible (e.g., ‘minimal’ & ‘not very long’—were categorized as 0 to 4 years).

## 3. Results

### 3.1. Poster Respondents and Use

Participation varied based on the event; the OIE research conference had the lowest participation (approximately 22%, or 84 out of approximately 380 academic, government, veterinary, and other animal-use attendees), while the other two events had higher participation (AGRI: 100% of all 38 students; SRVO: 82% or 37 out of 45 veterinarians).

Participant feedback about the poster format and research method was minimal and from the research conference (OIE) participants only. There was no negative feedback provided; eight participants specifically commented on its novelty and interactivity. Two participants contacted the research team following the event and requested permission to use the format in their own research. 

### 3.2. Natural Behaviour Importance Ranking 

**Figure 3:**
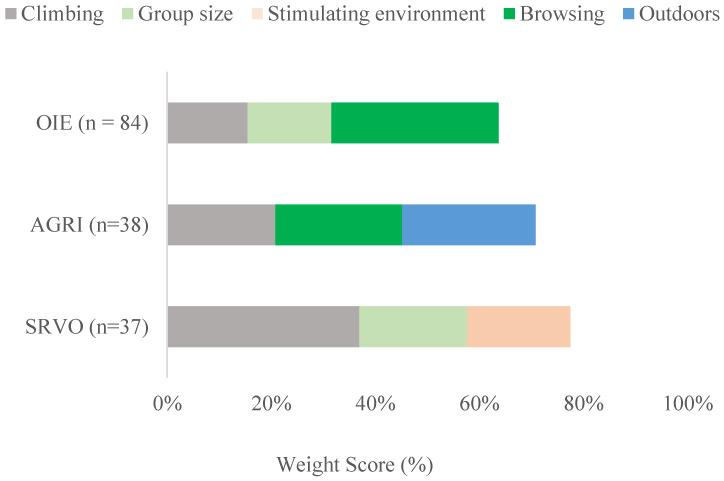
Ranked importance of opportunities for natural behavior expression in goats for the World Organisation for Animal Health World Conference on Animal Welfare (OIE), the AgriLeadership student site visit to AgResearch Ltd. (AGRI), and at the Small Ruminant Veterinarians of Ontario Annual Conference (SRVO). Importance was expressed as a weighted score, calculated as a percentage of the total responses. Participants could rank any of the eight provided opportunities according to their top three options.

Rankings from the three conferences are presented in Figure 3. The research conference (OIE) attendees ranked browsing opportunity highest overall (32.1%). Participants from South America (n = 5) ranked climbing opportunities as their top opportunity (43.3%), while participants from Africa (n = 9) ranked outdoor access as being equally important to browsing (29.6% for each). For the second-highest ranking, government/NGO (n = 10) and researchers (n = 13) selected climbing, veterinarians (n = 40), and ‘other’ occupations (n = 11) ranked appropriate group size, while academic/student (n = 10) occupations selected the opportunity for does and kids to interact. School children (AGRI) placed relatively equal importance across their top three behavioral opportunities; overall, outdoor space (25.7%) was important but was followed closely by browsing (24.3%) and climbing opportunities (20.8%). When stratified by experience with animals, students with no experience or those with only companion pets (n = 12), ranked browsing opportunities (26.4%) over outdoor space (22.2%). At the veterinary conference (SRVO), climbing opportunities were ranked highest (36.9%), followed by appropriate group size and dynamics (20.7%) and stimulating environment (19.8%); when these participants were stratified by experience with goats, those that had the most experience (20 years or more, n = 11), ranked browsing opportunities (16.7%) over stimulating environment (10.6%). 

## 4. Discussion

We piloted a simple, yet interactive data-gathering poster by deploying it in three different animal welfare contexts. This application was intended to demonstrate how the format could be used to generate indicative data representing stakeholder views. While we specifically collected participants’ opinions on conditions and behaviors that contribute positively to naturalness in goats, we caution that this was a proof-of-concept, and, therefore, the specific results are indicative only. We found that participation was high in a small group setting (e.g., our two examples with 45 or fewer attendees). For the larger conference, several factors could have contributed to the low participation, including poster location (e.g., not near the refreshments) and the size of the room limiting the number of people who could visit during the poster sessions. Furthermore, the actual poster session attendance could not be monitored at this conference; therefore, it is possible that some conference attendees never entered the poster session. 

### 4.1. The Interactive Poster Format

Academic posters are a popular way to present research findings in a quick and brief form. With an estimated 1.1 million poster presentations at conferences each year, they are second only to journal articles as a chosen form of research communication [16]. However, the information they convey is limited by the constraints of the medium, which cannot accommodate the amount of information contained in a journal article [17]. It is also limited by the fact that it is part of the ‘grey literature’ (i.e., literature not controlled by a publisher [16]). This limits its persistence in the academic record, unlike journal articles, which publishers provide access to, and which form the mainstream of material for libraries and archives. The element of the poster that is present in databases, libraries, and archives is generally limited to abstracts [5,16]. While the abstracts often are, posters are not subject to peer-review or other quality assurance processes. For these reasons, posters are often regarded as lower quality than other academic outputs [16,18]. These claims have been disputed [16,18], and we do not take a position on this matter. It is recognized that these issues ought to be addressed [16,18], and remedies for some of these issues have been proposed [5].

Our approach to the poster avoids many of these problems. Since the poster is now partly a data-gathering tool, and not a poster publishing results, it is not susceptible to criticisms that it is of lower quality and not subject to peer-review. As a data-gathering tool, it is a piece of human participant research if accompanied by ethical review. Furthermore, while ours did not specifically present data, it could easily serve to disseminate information in a structured way and then immediately and systematically gather input based on that information. This is one of the key goals of poster presentations that do not often occur at conferences [5]. The benefits aside, it is important to note that data-gathering is normally finite. The processes for gathering data using this poster are operational while it is being displayed, and it is not desirable for it to persist after display. The lack of persistence of a poster in the academic record is, therefore, benign (or beneficial) for the type of poster we are proposing.

When data gathering is facilitated by a ‘presenter’ of the poster (i.e., the researcher), as in our case, the form of the poster encourages interaction between the researcher and the audience [5,18], who may be participants in the research. The passive nature of poster presentations—that it is not accompanied by an oral presentation and physical interaction – has been identified as a key weakness of posters in knowledge transfer [17]. This interaction, if handled competently by the researcher, can help to ensure that the quality of the informed consent is high and that the participants have a good understanding of what is being asked of them in the research. This facilitates high-quality data gathering. 

Like any data-gathering involving the researcher, care must be taken to reduce the possibility of a systematic effect on participant responses that could bias or ‘herd’ the data, or a random effect that would increase the variability of responses [19,20]. Socially desirable responding (i.e., eliciting responses intended to be acceptable, rather than truthful) can be a problem for any human participant data gathering method, and this is particularly a concern if data is sensitive in nature [20]. Since data gathering is not private, there is an opportunity for the researcher to unconsciously influence the response of a participant [20]. The questions posed in this study were not sensitive, so an effect of this type is unlikely. We suggest follow-up work could allow participants to provide opinions prior to any interaction with the presenter, and then again following the discussion. We also recommend collecting data on whether participants provide answers before or after talking to the presenter, to allow testing of any presenter interaction effect. This application would also create a separate opportunity to test the efficacy of different presenters if applied across a larger cohort of participants and presenters. Another way to avoid the presenter effect is to carefully select the presenter(s) as someone that can establish a suitable rapport with participants [20] 

Furthermore, we suggest it may be possible to collect data without the researcher present, through using the poster to present information, and including a digital data collection method integrated into the presentation, instead of stickers. Recent developments such as ‘Digital Interactive Poster Presentations’ could be good candidates for this application, although their use in data collection has not to our knowledge been explored [21,22]. Combining these with suitably adapted online audience-response systems (e.g., Mentimeter; Stockholm, Sweden), which are used in technology-enhanced teaching and learning [23,24], may allow data collection to occur anonymously and without the researcher present. Use of such a system would allow the collection of data to be automated, anonymous (if desired) and immediate; nonetheless, it would be dependent on the existence of reliable infrastructure (e.g., accessible internet) in the poster deployment location to work effectively.

Interestingly, at the one event where we made participation possible without the presenter present (OIE), the interaction was minimal (less than 10% of the responses occurred during this time). This aside, data gathering in an interpersonal setting is not new in research. Critical reflection on the data produced, and the means for gathering it, which includes the researcher’s role (i.e., a process termed ‘functional reflexivity’) is a well-developed and established component of successful interactive qualitative or quantitative research [25]. It ought, therefore, to be applied in interactive data gathering posters to address this issue.

A further effect of the public nature of responding to the poster is that other participants may be influenced by the responses of those before them, raising another avenue for social desirability [20] or other social factors, to potentially influence responses. Of course, this is a possibility in focus group interviews as well; therefore, the tools of moderating among participants in a group may also be applied to our tool to reduce influence [26]. Indeed, in the two smaller events (AGRI and SRVO), the presenter gave out stickers and told respondents to think about their ranking prior to approaching the poster, in an effort to not only expedite the process but also to moderate and minimize the influence from other participants’ answers. Alternatively, allowing others to know the responses of other participants may be viewed as a desirable aspect of the data-gathering method, allowing participants to respond to information gathered, as well as contributing their own. Although not using our poster method, this latter approach has been used successfully with small groups in ‘virtual town halls’ to gather data on public beliefs about animal welfare [27]. 

A virtue of this new poster format is that it is highly flexible. It can be applied to a wide range of disciplines and settings. If presenter attendance at the poster is not possible, then data gathering can be achieved by incorporating a secure data collection device into the poster (e.g., feedback collection buttons after airport security screening). Collection can, therefore, occur anywhere information (e.g., a poster) can be displayed. This may also make data collection less burdensome on researchers and allow data collection for longer periods and at more locations.

A problem with this method of data collection is that there may be bias in the sample. For example, if data is collected at an academic conference, it may not be diverse in some demographic variables and may share common interests that are relevant for the poster topic. More generally, since responding is voluntary, and likely in part due to a participant being interested in the poster, or their willingness to engage in an interpersonal setting, this may result in a sample being unrepresentative of the general population. This weakens the generalizability of the data and limits the extent of the generalizable empirical claims made. There is nothing in the method that prevents screening of participants based on inclusion and exclusion criteria to address this generalizability issue; however, provided demographic information is obtained and reported along with the results, improper generalizations will be avoided. If demographic information is also collected elsewhere, such as in the pool of all conference attendees, this can allow a further inference to be made about the representativeness or otherwise of the sample with respect to that larger pool, if desired. Moreover, it is often useful to poll a small population to find out their views regardless of any general inferences; this is often the case with formats like focus-groups, and the gathering of qualitative data through this method is very valuable [26].

### 4.2. Stakeholder Views of Contributors to Goat Welfare

As an exemplar of the methodology, the poster served to gather information from 3 different cohorts of event participants about their views on naturalness in goats. ‘Natural living’ or ‘naturalness’ constitutes one of three broad and influential accounts of animal welfare [6,28]. According to this account, an animal is benefited intrinsically by living in accordance with its nature. Expression of natural behaviors and other natural functions, therefore, increases an animal’s welfare. Any environment housing animals that allows the opportunity for naturalness is, to that extent, good for promoting animal welfare on this view [29].

Determining what are natural behaviors for an animal is not straightforward. If by natural we mean adaptive (i.e., fitness-enhancing in an evolutionary sense) behavioral traits, this is plastic and varies between animals, and within an animal, on the basis of a wide range of factors [30]. Moreover, some of these behaviors may harm animals in various ways, putting pressure on the claim that opportunities for expression of natural behavior necessarily increases welfare [30]. Nevertheless, there is good evidence for some natural behaviors, having a range of benefits, such as goats’ preferences for climbing in elevated, rocky terrains [10]. We caution that the data collection performed in this study was intended to demonstrate a proof-of-concept of the poster format. Therefore, we cannot make robust comparisons across cohorts. We have, however, demonstrated that when applied in different settings, our tool gathered some consistent opinions on which behaviors are the best exemplars of naturalness.

The results of this research tool informed our research program (e.g., [10,31]). Empirical research such as this can be important because the views of the public will inform their consumptive habits, and the disjunction between public views and those of farmers or others can cause commercial problems for the animal industry [32]. Democracy requires public support for policies or practices that aim to promote welfare in animal care and use industries [15,33]. Knowing what different social groups perceive as indicators of good welfare states for particular animal types is important for policy development and agricultural practice. 

## 5. Conclusions

Interactive data-gathering posters are an ethically valuable tool in animal welfare research. They provide a convenient, innovative tool for gathering empirical information about beliefs about animal welfare and the concrete reality that ethical and empirical concepts like animal welfare operate in, and are informed by [34,35]. This can allow the continual responsiveness of animal welfare science and animal ethics theory to the social context in which humans interact with animals, generating socially informed and, therefore, socially robust results [36,37]. Continued use and refinement of the interactive poster method, including testing out electronic versions, with and without the presence of presenters, and in various demographical contexts, will promote evolution of the classic academic poster presentation. The results gathered will improve the grounding of science and ethics in social reality and social participation for the benefit of both animals and the humans who care for them.
